# Polyp characterization using deep learning and a publicly accessible polyp video database

**DOI:** 10.1111/den.14500

**Published:** 2023-01-18

**Authors:** Rawen Kader, Anton Cid‐Mejias, Patrick Brandao, Shahraz Islam, Sanjith Hebbar, Juana González‐Bueno Puyal, Omer F. Ahmad, Mohamed Hussein, Daniel Toth, Peter Mountney, Ed Seward, Roser Vega, Danail Stoyanov, Laurence B. Lovat

**Affiliations:** ^1^ Wellcome/EPSRC Centre for Interventional and Surgical Sciences University College London London UK; ^2^ Division of Surgery and Interventional Sciences University College London London UK; ^3^ Gastrointestinal Services University College London Hospital London UK; ^4^ Odin Vision Ltd London UK

**Keywords:** artificial intelligence, colonic polyp, colonoscopy, colorectal neoplasm, deep learning

## Abstract

**Objectives:**

Convolutional neural networks (CNN) for computer‐aided diagnosis of polyps are often trained using high‐quality still images in a single chromoendoscopy imaging modality with sessile serrated lesions (SSLs) often excluded. This study developed a CNN from videos to classify polyps as adenomatous or nonadenomatous using standard narrow‐band imaging (NBI) and NBI‐near focus (NBI‐NF) and created a publicly accessible polyp video database.

**Methods:**

We trained a CNN with 16,832 high and moderate quality frames from 229 polyp videos (56 SSLs). It was evaluated with 222 polyp videos (36 SSLs) across two test‐sets. Test‐set I consists of 14,320 frames (157 polyps, 111 diminutive). Test‐set II, which is publicly accessible, 3317 video frames (65 polyps, 41 diminutive), which was benchmarked with three expert and three nonexpert endoscopists.

**Results:**

Sensitivity for adenoma characterization was 91.6% in test‐set I and 89.7% in test‐set II. Specificity was 91.9% and 88.5%. Sensitivity for diminutive polyps was 89.9% and 87.5%; specificity 90.5% and 88.2%. In NBI‐NF, sensitivity was 89.4% and 89.5%, with a specificity of 94.7% and 83.3%. In NBI, sensitivity was 85.3% and 91.7%, with a specificity of 87.5% and 90.0%, respectively. The CNN achieved preservation and incorporation of valuable endoscopic innovations (PIVI)‐1 and PIVI‐2 thresholds for each test‐set. In the benchmarking of test‐set II, the CNN was significantly more accurate than nonexperts (13.8% difference [95% confidence interval 3.2–23.6], *P* = 0.01) with no significant difference with experts.

**Conclusions:**

A single CNN can differentiate adenomas from SSLs and hyperplastic polyps in both NBI and NBI‐NF. A publicly accessible NBI polyp video database was created and benchmarked.

## INTRODUCTION

Approximately 60% of polyps detected during colonoscopy are diminutive (≤5 mm), with two‐thirds of these adenomas.^1^, ^2^ These harbor a very low risk for developing colorectal cancer, yet histological diagnosis represents a significant burden to histopathologists with associated costs to health‐care systems.^2^, ^3^ Implementation of endoscopic technology for ‘resect and discard’ of diminutive adenomas is required to meet the preservation and incorporation of valuable endoscopic innovations (PIVI)‐1 threshold, which is ≥90% concordance in postpolypectomy surveillance intervals when comparing the combination of optical diagnosis (OD) for diminutive adenomas with histopathology assessment of all other polyps against decisions based solely on histopathology evaluation of all identified polyps.^4^, ^5^, ^6^ PIVI‐2 (‘diagnose and leave’) requires a negative predictive value (NPV) of ≥90% for diminutive adenomas in the rectosigmoid.^2^ Whilst many endoscopists have adopted ‘diagnose and leave’, adoption of ‘resect and discard’ has been less successful, partly due to studies demonstrating that nonexpert endoscopists fall short of the PIVI‐1 threshold, with predominately expert endoscopists in academic centers surpassing it.^6^, ^7^

Recent years have demonstrated the potential of deep‐learning to characterize polyps using narrow‐band imaging (NBI), resulting in optimism that it can support nonexpert endoscopists to achieve PIVI.^8^, ^9^, ^10^, ^11^ However, most computer‐aided diagnosis (CADx) models are trained using a single NBI modality, typically NBI‐near focus (NF), restricting their applicability to newer Olympus endoscopes which include NBI‐NF. Furthermore, most are developed with high‐quality retrospective still images captured by expert endoscopists and limited to a few images per polyp, often excluding sessile serrated lesions (SSLs) altogether.^11^, ^12^ Little is also known of their generalizability to different clinical settings due to the limited number of public datasets available for CADx.^13^

This study aims to develop an artificial intelligence (AI) model to characterize polyps, including SSLs, as adenoma or nonadenoma in both standard NBI and NBI‐NF and to create a publicly accessible NBI polyp video database.

## METHODS

### Data collection

Unaltered endoscopy videos were prospectively collected from April 2019 to February 2021 at University College London Hospital to develop various AI algorithms for colonoscopy. Procedures used a high‐definition endoscope (CF‐HQ290L, CF‐HQ260L; Olympus, Tokyo, Japan), an Olympus EVIS LUCERA‐CV290(SL) processor and were recorded with a high‐definition video recorder (UR‐4MD; TEAC, Tokyo, Japan). CF‐HQ260L endoscopes provide standard NBI, whilst CF‐HQ290L also includes NBI‐NF, a 40‐fold magnification of standard NBI (figure S1 in Appendix S1).

**Figure 1:**
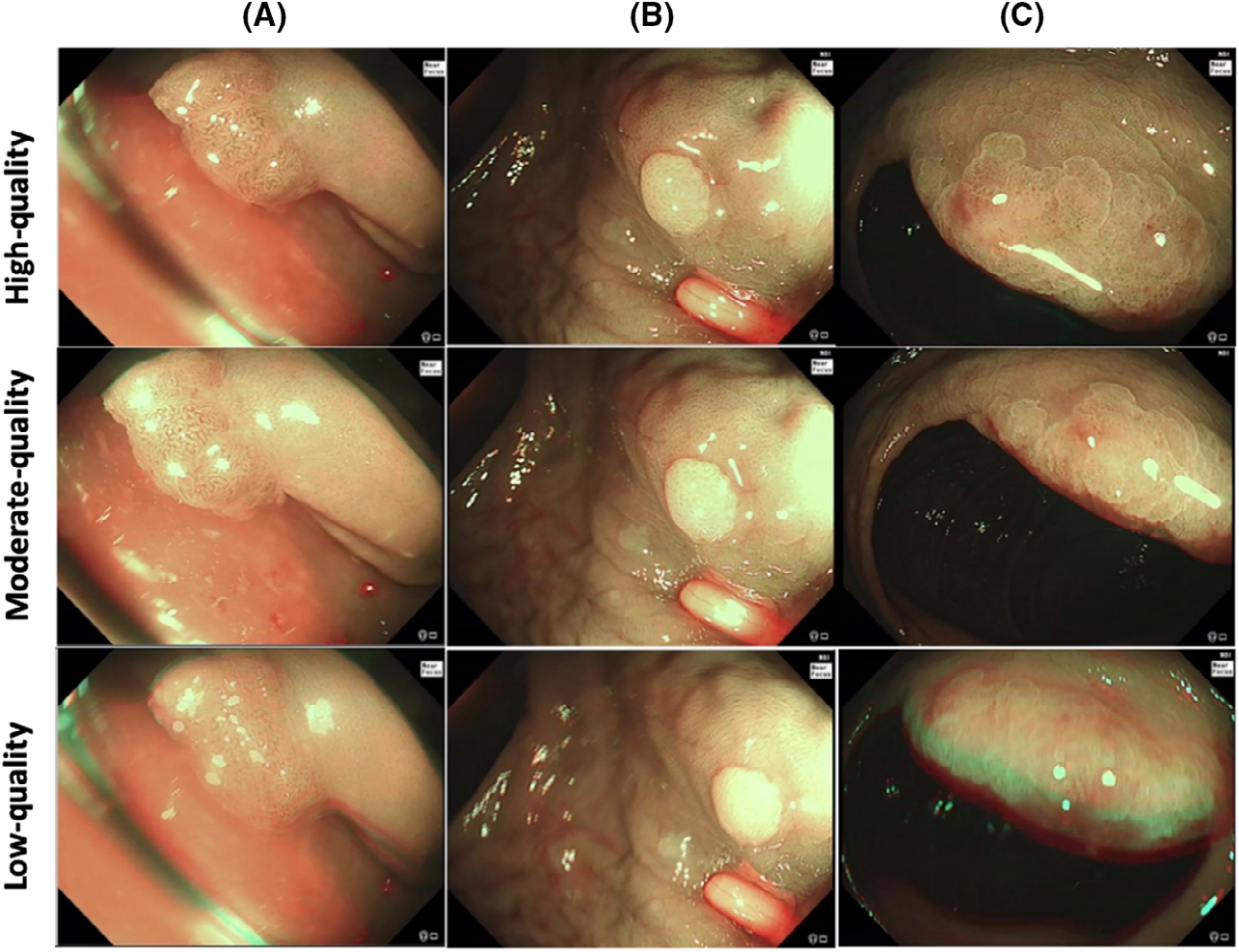
Illustration of image quality annotation. Video frames from the same polyp classified as high (top row), moderate (middle row), and low quality (bottom row). (A) Tubular adenoma – image‐related blurriness and artifact present in the low‐quality image. (B) Hyperplastic polyp – significant halation present in the low‐quality image. (C) Sessile serrated lesion – image‐related blurriness present in the low‐quality image.

All patients were eligible for inclusion, and procedures were carried out as per standard of care. The institute's expert gastrointestinal histopathology department classified polyps according to the latest World Health Organization guidelines.^14^ Polyps in NBI were also optically assessed to confirm consistency with the histological diagnosis. OD inconsistent with the histopathology, lesions with benign diagnoses such as lymphoid follicles, and adenocarcinomas were excluded. The remaining polyps were categorized into adenomas (tubular, tubulovillous, and villous) and nonadenomas (hyperplastic, SSLs, and traditional serrated adenomas [TSA]). The location, Paris classification, and size were also collected for each polyp. Moderate and high‐quality frames were annotated, as outlined in Appendix S1, and referenced as the ground truth (Fig. 1).

### Databases

Two databases were curated using the methodology outlined above.

#### Database I

**Figure 2:**
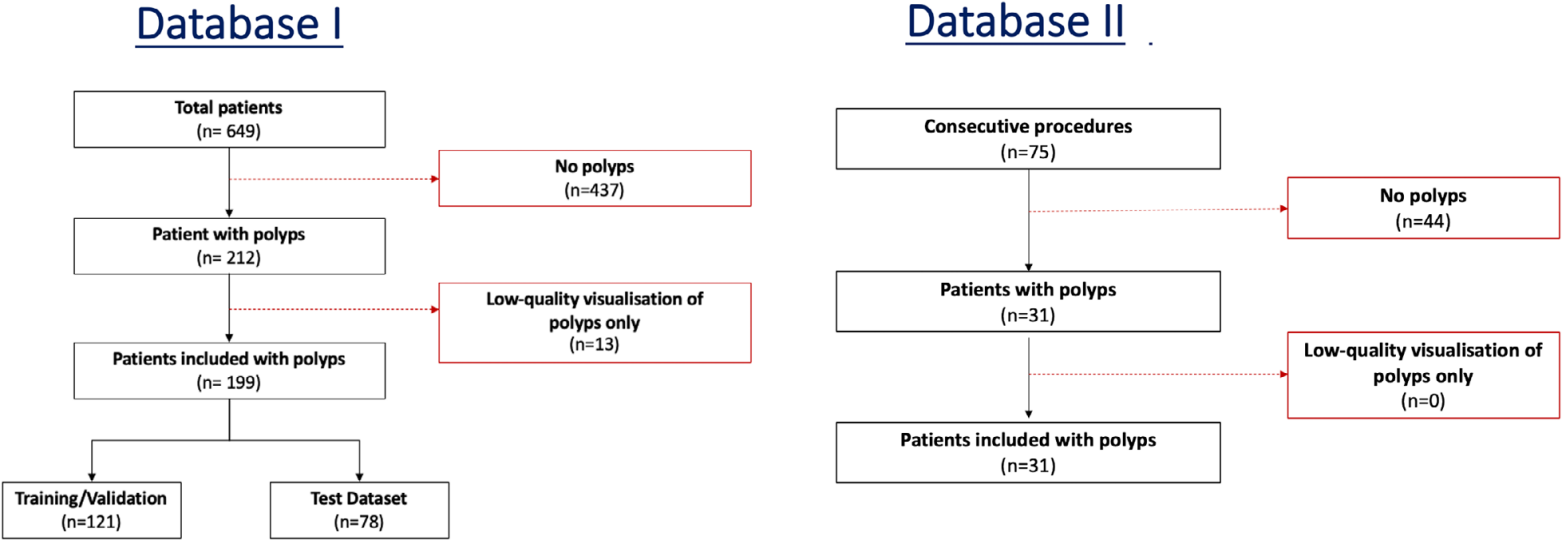
Patient flowchart. Patient flowchart for curating database I (training, validation, and test‐set I) and database II (test‐set I). For database I, 13 patients with narrow‐band imaging polyps were excluded due to low‐quality visualization of polyps throughout the video sequence.

Database I was intended for training and initial testing (test‐set I) of the convolutional neural network (CNN). Patients were recruited from April 2019 to November 2020, with periodic pauses in recruitment secondary to COVID. Patients were not screened beforehand, as all patients were eligible for inclusion. All procedures recorded during this timeframe were reviewed for inclusion. A total of 437 patients had no polyps, and 212 had histologically confirmed polyps in NBI (Fig. 2). Thirteen of these 212 patients were excluded due to low‐quality visualization of polyps throughout the video (e.g., dark images throughout or overlying colonic content not washed from the polyp surface).^15^

There were 386 polyps (244 adenomas, 83 SSLs, 58 hyperplastic, and one TSA) in the remaining 199 patients and 31,152 moderate/high‐quality frames. The polyps were separated into training (~50%), validation (~10%) and testing set (40%) with no overlap of data or patients (Table 1). To reduce patient bias, data were separated whereby patients with more than five polyps represented <5% of the total patients and 15% of the total polyps in the training and validation dataset.

**Table 1:** Baseline characteristics of databases I and II

Variable	Database I			Database II	Total
	Training	Validation	Test‐set I	Test‐set II (WEISS database)	
Total number of patients	107	14	78	31	230
Total number of polyps	197	32	157	65	451
Histology					
Adenoma					
Tubular HGD	1	0	0	3	4
Tubular LGD	124	16	91	34	265
Tubulovillous LGD	5	3	4	2	14
Total	130	19	95	39	283
Nonadenoma					
Hyperplastic	21	2	35	16	74
SSL with dysplasia	1	1	1	0	3
SSL without dysplasia	44	10	26	10	90
Traditional serrated adenoma	1	0	0	0	1
Total	67	13	62	26	168
Location					
Cecum	24	8	18	13	63
Ascending colon/hepatic	59	5	56	13	133
Transverse	38	4	21	12	75
Descending colon/splenic	28	5	25	9	67
Rectosigmoid	48	10	37	18	113
Morphology					
Ip	5	1	5	1	12
Is and Isp	106	15	76	26	223
IIa	81	16	71	36	204
IIa and IIc	0	0	1	0	1
IIb	1	0	1	2	4
LST‐NG	3	0	0	0	3
LST‐G	1	0	3	0	4
Size					
≤5 mm	100	13	111	41	265
>5 mm or ≤10 mm	69	12	37	13	131
>10 mm	28	7	9	11	55

G, granular; HGD, high‐grade dysplasia; LGD, low‐grade dysplasia; LST, lateral spreading tumor; NG, nongranular; SSL, sessile serrated lesion; WEISS, Wellcome/EPSRC Centre for Interventional and Surgical Sciences.

The CNN was trained and validated with 229 polyp videos (113 diminutive), consisting of 149 adenomas, 56 SSLs, 35 hyperplastic and one TSA polyp from 121 patients and 16,832 frames (67,999 diminutive). Test‐set I consisted of 157 polyps (111 diminutive) from 78 patients and 14,320 frames (10,383 diminutive). These polyps are separate from those in the training and validation dataset.

#### Database II – test‐set II (WEISS database)

To create a robust publicly accessible dataset (test‐set II), NBI polyps were collected from 75 consecutive cases between December 2020 and February 2021. Forty‐four patients were found to have no polyps, and 31 patients had histologically confirmed polyps in NBI. The test‐set includes a total of 65 polyps (41 diminutive) and 3317 moderate/high‐quality frames. To benchmark the public dataset, we compared the CNN performance to three expert national bowel cancer screening program (BCSP) accredited colonoscopists (adenoma detection rate >45%) and three nonexpert colonoscopists accredited for independent colonoscopy by the UK Joint Advisory Group on gastrointestinal endoscopy. Further details of this benchmarking process are available in Appendix S1.

### Developing the CNN

**Figure 3:**
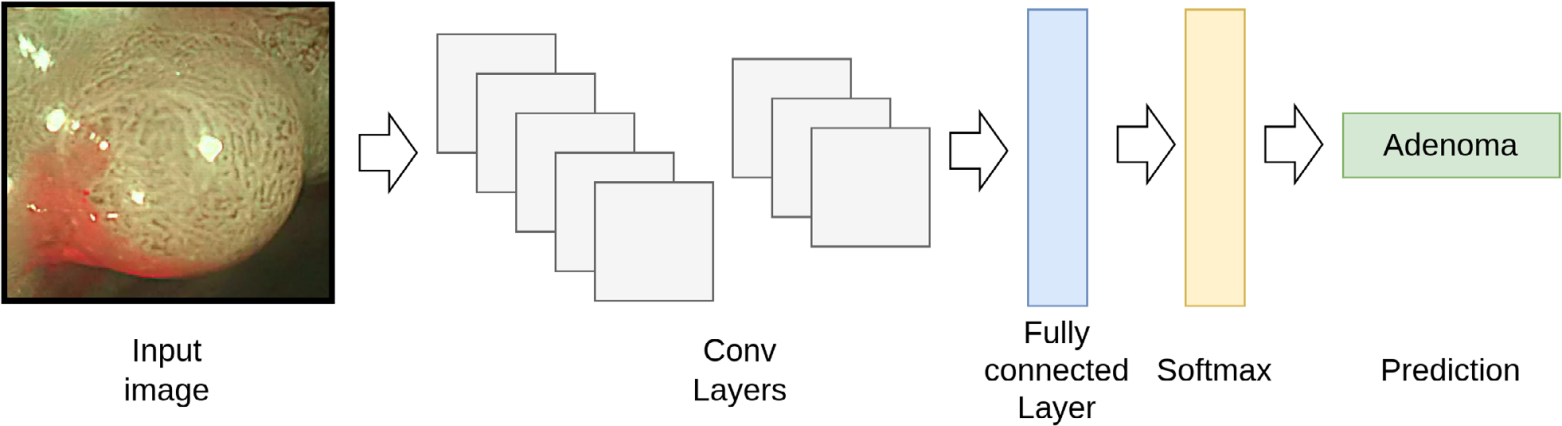
Example of how a convolutional neural network prediction is obtained. (1) An image is passed through a series of concatenated convolutional layers that output a feature vector. (2) The vector is passed to a fully connected layer that processes it into two values (logits). (3) The logits are processed by a Softmax function that returns a prediction score between 0 and 1 that, depending on its value, is interpreted as adenoma or nonadenoma.

We developed a CNN to characterize polyps as adenoma or nonadenoma. In brief, an image is passed through a series of concatenated convolutional layers that output a feature vector (Fig. 3). The vector is passed to a fully connected layer that processes it into two values (logits). The logits are processed by a Softmax function that returns a prediction score between 0 and 1 that, depending on its value, is interpreted as adenoma or nonadenoma. A threshold of 0.6 was chosen as the operating point resulting in a CNN prediction score of 0.6–1.0 characterizing as adenoma and less than 0.6 as nonadenoma. The prediction score does not include a confidence threshold; therefore, all CNN outputs are treated as high confidence with no predictions rejected. Further algorithm development details are available in Appendix S1.

### Statistical analysis

The CNN performance for each test‐set was measured on a per‐frame and per‐polyp level. Analyses included polyps of all sizes, diminutive polyps, each polyp category, each NBI modality, and performance compared to PIVI thresholds. For the per‐polyp analysis, we analyzed the proportion of annotated frames for each polyp that the CNN correctly characterized. A correct diagnosis was defined as ≥50% of frames correctly characterized by the CNN. For test‐set II, we compared the accuracy of the CNN to the expert and nonexpert endoscopists for all polyps, high‐confidence (HC) diagnoses, diminutive polyps, and HC diagnoses of diminutive polyps.

The agreement between the CNN‐predicted and histological diagnoses was evaluated using sensitivity, specificity, accuracy, Cohen's kappa coefficient, and area under the curve (AUC). Further information on calculation of these metrics is available in Appendix S1. The data were summarized using descriptive statistics. Categorical data are presented with count and percentages, parametric continuous data consist of means and standard deviation, and nonparametric continuous data as medians with interquartile range. For evaluation and comparison of the endoscopists and CNN accuracy in test‐set II, we used a two‐sample test of proportions, used in combination with percentile bootstrap method based on 100 resamples with associated 95% confidence intervals (CI) presented. *P*‐values of ≤0.05 were deemed statistically significant. Statistical analyses were performed using Python (version 3.7.4; Wilmington, DE, USA) and Stata (version 15.1; College Station, TX, USA).

## RESULTS

### Test‐set I

#### All polyps

**Figure 4:**
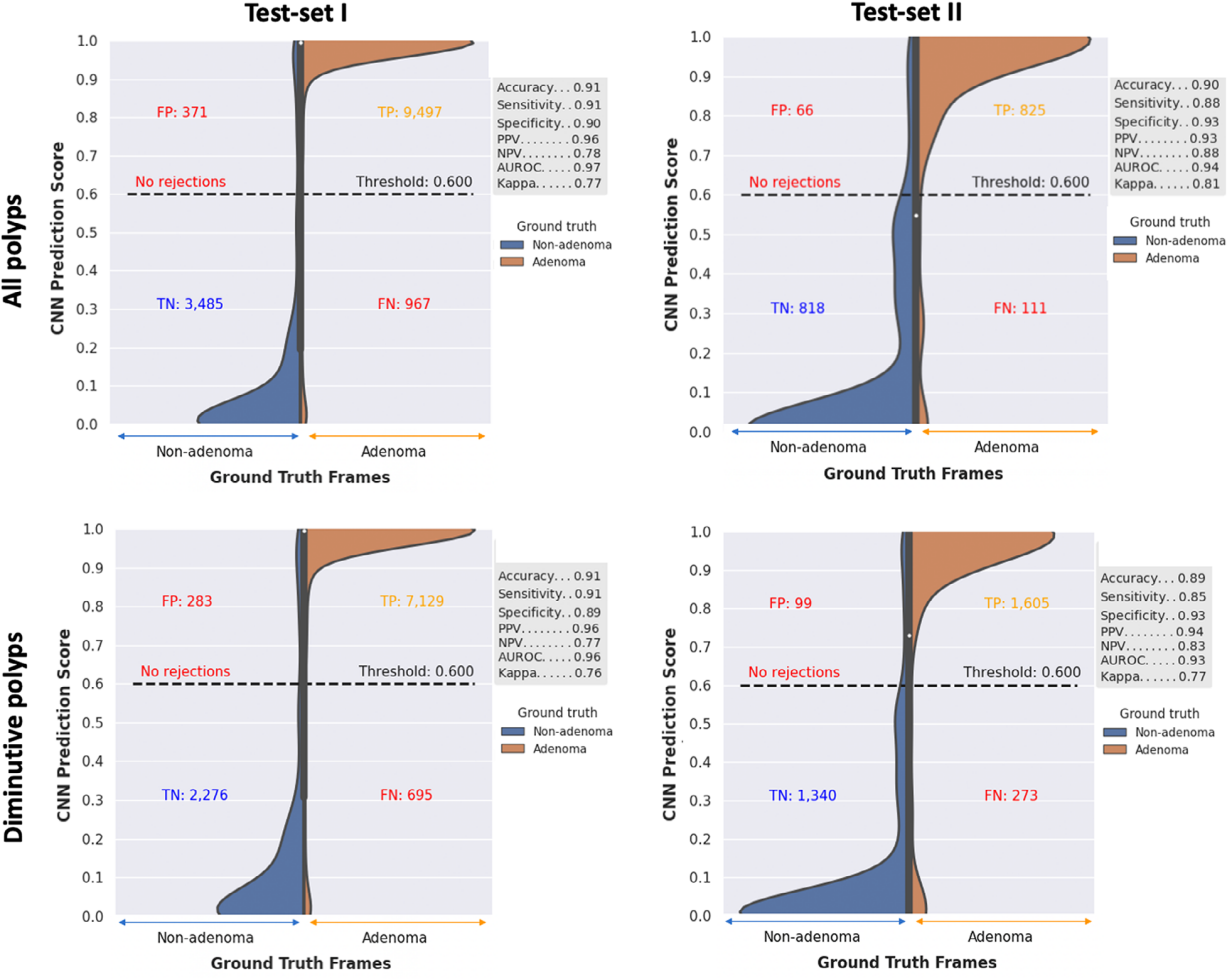
Convolutional neural network (CNN) per‐frame results. Results demonstrated separately for polyps of all size and diminutive polyps (≤5 mm) in test‐sets I and II. CNN prediction score of 0.6–1.0 characterizes a polyp as adenomatous and less than 0.6 as nonadenomatous. AUROC, area under the receiver operating characteristic curve; FN, false negative; FP, false positive; NPV, negative predictive value; PPV, positive predictive value; TN, true negative; TP, true positive.

**Figure 5:**
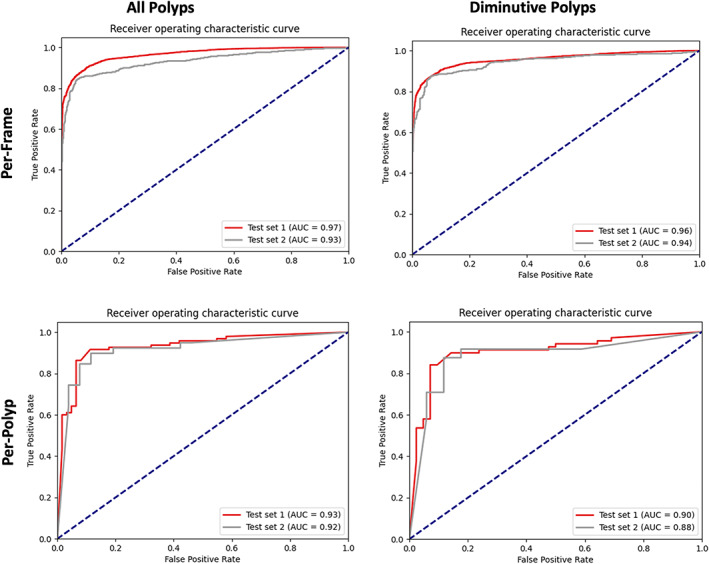
Convolutional neural network area under the curve (AUC) results. Per‐frame and per‐polyp results demonstrated for test‐set I and test‐set II. Results are presented separately for polyps of all sizes and diminutive polyps.

From 157 polyps, the CNN characterized adenomas with a sensitivity, specificity, and AUC of 90% or above in the per‐frame (Fig. 4) and per‐polyp analysis (Table 2; Fig. 5). The CNN misclassified 13 polyps: eight adenomas, three SSLs and two hyperplastic.

**Table 2:** Convolutional neural network per‐polyp results

	Polyp size	Number of polyps				Results (%)			
		Total	Adenomas	Hyperplastic	SSL	Sensitivity	Specificity	Accuracy	AUC
Test‐set I	All polyps	157	95	35	27	91.6	91.9	91.7	93.0
	≤5 mm	111	69	31	11	89.9	90.5	90.1	90.0
Test‐set II	All polyps	65	39	16	10	89.7	88.5	89.2	91.7
	≤5 mm	41	24	14	3	87.5	88.2	87.8	88.2

Results demonstrated separately for test‐set I and test‐set II. This includes separate analysis for polyps of all sizes and diminutive polyps for each test‐set.

AUC, area under the curve; SSL, sessile serrated lesion.

#### Diminutive polyps

From 111 diminutive polyps in test‐set I, the CNN characterized adenomas with a sensitivity, specificity, and AUC of 89% or above in the per‐frame and per‐polyp analysis. Eleven of the aforementioned 13 polyps misclassified were diminutive: seven adenomas, two SSLs, and two hyperplastic. The CNN sensitivity and specificity were higher in NBI‐NF compared to NBI: 89.4% vs. 85.3% and 94.7% vs. 87.5% (Table 3).

**Table 3:** Results for each imaging modality (narrow‐band imaging [NBI] and NBI‐near focus [NF])

	Image modality	Diminutive polyps				Results (%)		
		Total	Adenomas	Hyperplastic	SSL	Sensitivity	Specificity	Accuracy
Test‐set I	Overall	111	69	31	11	89.9	90.5	90.1
	NBI‐NF	66	47	12	7	89.4	94.7	90.9
	NBI	58	34	20	4	85.3	87.5	86.2
Test‐set II	Overall	41	24	14	3	87.5	88.2	87.8
	NBI‐NF	31	19	11	1	89.5	83.3	87.1
	NBI	22	12	8	2	91.7	90.0	90.9

Results shown are for diminutive polyps for each imaging modality. ‘Overall’ result includes all polyps in each respective test‐set. The separate NBI and NBI‐NF results only include polyps with frames from each respective imaging modality.

SSL, sessile serrated lesion.

### Test‐set II

#### All polyps

From 65 polyps in test‐set II, the CNN sensitivity, specificity, and AUC in the per‐polyp analysis were 88% or above (Table 2). In the per‐frame analysis, it was 86% or above. The CNN misclassified seven polyps: four adenomas and three hyperplastic.

#### Diminutive polyps

In the per‐polyp analysis, the overall CNN sensitivity, specificity, and AUC were 87.5%, 88.2%, and 88.2%, respectively. In the per‐frame analysis, these were 88.1%, 92.5%, and 94.5%. Three of the aforementioned seven polyps misclassified were diminutive: two adenomas and one hyperplastic. The CNN correctly classified diminutive polyps as adenoma or nonadenoma with an accuracy greater than 88% for each polyp category (adenoma, hyperplastic, and SSL). The CNN sensitivity to characterize diminutive adenomas was 89.5% in NBI‐NF and 91.7% in NBI.

#### Benchmarking

For test‐set II, the CNN was significantly more accurate than nonexperts (Table 4). This was true for the difference in accuracy of all polyps irrespective of OD confidence (13.8% [95% CI 3.2–23.6], *P* = 0.01), HC diagnoses (11.0% [95% CI 0.6–21.3], *P* = 0.04), diminutive polyps irrespective of OD confidence (15.4% [95% CI 4.5–26.7], *P* = 0.01), and HC OD of diminutive polyps (12.2% [95% CI 0.0–24.1], *P* = 0.05). In all analyses, the accuracy of the CNN was numerically higher than the expert BCSP endoscopists, but there was no significant difference.

**Table 4:** Benchmark experiment for test‐set II: convolutional neural network (CNN) vs. endoscopists

Accuracy (%)	Nonexperts	Experts	CNN	Difference between accuracy of CNN and endoscopists (95% CI)	
				CNN vs. nonexperts	CNN vs. experts
All polyps					
All diagnoses	75.4 (67.7, 80.0) 147/195	82.6 (77.4, 86.2) 161/195	89.2 (79.1, 95.6) 58/65	13.8 (3.2, 23.6) ***P* = 0.01**	6.7 (−0.6, 15.7) *P* = 0.20
HC OD	78.3 (72.8, 83.2) 144/184	85.2 (81.9, 92.3) 155/182	89.2 (79.1, 95.6) 58/65	11.0 (0.6, 21.3) ***P* = 0.04**	4.1 (−2.4, 15.6) *P* = 0.35
Diminutive polyps					
All diagnoses	72.4 (64.2, 79.7) 89/123	79.7 (74.0, 86.2) 98/123	87.8 (73.8, 95.9) 36/41	15.4 (4.5, 26.7) ***P* = 0.01**	8.1 (−2.1, 24.1) *P* = 0.15
HC OD	75.7 (67.0, 82.6) 87/115	83.8 (79.3, 92.8) 93/111	87.8 (73.8, 95.9) 36/41	12.2 (0.0, 24.1) ***P* = 0.05**	4.0 (−9.6, 11.6) *P* = 0.20

Comparison of the accuracy of the CNN compared to expert and nonexpert endoscopists. Separate analysis performed for polyps of all sizes (“all polyps”), high‐confidence diagnoses of “all polyps”, diminutive polyps, and high‐confidence diagnoses of diminutive polyps. Statistically significant *P*‐values are highlighted in bold.

CI, confidence interval; HC, high confidence; OD, optical diagnosis.

### PIVI thresholds

For test‐set I (111 diminutive polyps) and test‐set II (41 diminutive polyps), there was 97% and 94% respective concordance between the CNN‐predicted colonoscopy surveillance intervals compared to histology‐derived intervals using the US Multi‐Society Task Force guidelines (Table 5).^16^ The NPV to diagnose diminutive rectosigmoid adenomas was 95% for test‐set I and 90% for test‐set II. For diminutive rectosigmoid polyps, one adenoma was misclassified in each test‐set, two hyperplastic polyps in test‐set I and one hyperplastic polyp in test‐set II.

**Table 5:** Convolutional neural network (CNN) performance for PIVI‐1

	Incorrect surveillance interval		Concordance between CNN‐predicted and histology‐derived surveillance interval (%)
	Histology‐derived	CNN‐predicted	
Test‐set I	10 years	7–10 years	97.0
	5–10 years	7–10 years	
Test‐set II	10 years	7–10 years	94.4

Patients' CNN‐predicted surveillance intervals using a ‘resect and discard’ strategy for diminutive adenomas compared to the ground truth histology‐derived surveillance intervals using the US Multi‐Society Task Force guidelines (PIVI‐1).

## DISCUSSION

In this study, a CNN was developed and tested to differentiate adenomas from SSL and hyperplastic polyps in standard NBI and NBI‐NF using a total of 451 polyp videos and 34,469 video frames. To our knowledge, this is the largest dataset in the literature of polyp videos and frames annotated using NBI. In the per‐polyp analysis, the CNN characterized diminutive adenomas in each test‐set with a sensitivity and specificity greater than 87% and generalized well to both standard NBI and NBI‐NF. Surprisingly, standard NBI was more accurate than NBI‐NF for test‐set II (89.7% vs. 87.5%), which may reflect the difficulty of polyps as not all polyps were visualized in both modalities. For the benchmarking of the publicly accessible test‐set II, the CNN was significantly more accurate than nonexpert endoscopists with no significant difference to expert endoscopists. The CNN in this study also achieved PIVI thresholds. This level of performance could potentially be helpful in improving the generalizability of the ‘resect and discard’ strategy. However, our results should be interpreted with caution as clinical trials are required, with appropriate statistical powering, to evaluate PIVI when the endoscopists' OD is supported with the CNN.

Several studies have demonstrated the potential of CADx to characterize polyps in NBI.^10^, ^11^, ^12^ However, these models have been primarily developed in a single NBI modality, typically NBI‐NF, and usually apply a confidence threshold when trained with both.^8^, ^9^, ^10^, ^11^ The former restricts its application to hospitals with newer generation Olympus endoscopes that include NBI‐NF but older generation Olympus endoscopes, which are limited to standard NBI, remain in circulation. The latter reduces the number of polyps it can support endoscopists to OD, with low‐confidence CADx diagnoses previously reported as high as 28%.^17^ The advantage of our CNN is that it operates without a confidence threshold and is applicable to both NBI imaging modalities, thereby increasing its usability. It was also trained and tested with video frames which are thought to be more reflective of “real‐world” quality images compared to still images extracted from endoscopy reports.^18^

The classification we used (adenoma vs. nonadenoma) allows the implementation of both PIVI strategies. As suggested by experts,^4^ diminutive adenomas throughout the colorectum would be ‘resect and discarded’ and diminutive rectosigmoid serrated polyps (nonadenomas) would be left in situ given the low prevalence of diminutive SSLs in this region,^5^ whilst diminutive serrated polyps proximal to this would be submitted to histopathology for differentiation of SSL and hyperplastic polyps. A multitier classification that separates each polyp category would be of greater clinical benefit. However, such a system requires a large training dataset, as evidenced in a recent single‐center study where sensitivity and specificity for differentiating SSLs from hyperplastic polyps were 80.8% and 62.1%, respectively.^19^

Our CNN achieved favorable results without using a confidence threshold despite a smaller sample size of polyps for training than other models. A plausible explanation is that annotating high‐ and moderate‐quality video frames compensated for the modest number of polyps.^20^ CADx models are often developed using retrospective still images from endoscopy reports of expert endoscopists, but this inherently limits the data to one or a few images per polyp. These models may also generalize less well to nonexpert endoscopists, who may be less likely to reproduce the high‐quality images of experts.^21^ An alternative approach is to train CADx models with successive video frames. Whilst this simplifies the clinical workflow by forgoing the need to activate the CADx manually, it can result in lower performance due to requiring the model to incorporate low‐quality frames in its assessment.^19^

Annotating high‐ and moderate‐quality frames also allowed us to identify that the orientation that a polyp is visualized, irrespective of adequate visualization to the human eye, can influence the CNN's performance. Over three‐quarters of polyps in each test‐set were correctly characterized in at least 80% of frames annotated, but the performance was more variable for 15% of polyps in test‐set I and 11% in test‐set II, with only 50–79% of annotated frames correct. For CADx to bridge the OD accuracy of experts and nonexperts, it should be consistent in its characterization in various views; however, this is poorly understood as CADx models are usually evaluated with only one or a few high‐quality images per polyp. If similar results are reproduced in other studies, future research should identify the optimal number of images required for CADx to provide a consistent characterization without significantly prolonging the procedural time.

Our CNN performed favorably compared to several other CADx systems using NBI, but direct comparison is meaningless without assessing with the same test‐set. Publicly accessible datasets for CADx are essential to enable this comparison. They also advance research by helping to overcome barriers such as the generalizability of CADx models to new clinical settings.^22^ At the time of writing, there are five publicly accessible datasets for CADe, but only two in optical chromoendoscopy for CADx.^23^ The developers of the CADx datasets should be commended, although they are not without limitations. Polyp videos in the Depeca dataset were recorded with a resolution of 768 × 576.^24^ This is lower than that encountered in clinical practice, introducing a degree of bias. The PICCOLO dataset is limited to still images, does not differentiate NBI frames from NBI‐NF, and has fewer polyps than ours.^25^ The WEISS database is a robust clinician‐developed publicly accessible database for CADx, consisting of 65 polyp videos in NBI and NBI‐NF in a minimum resolution of 1920 × 1072 and includes SSLs diagnosed from an institute with expertise in gastrointestinal histopathology. We also benchmarked the database to facilitate comparison with other CNNs.

There are several limitations of this study. First, data is limited to a single center and endoscopy system (Olympus), and further evaluation with external datasets is warranted. Further publicly accessible datasets, such as the WEISS database, would help to overcome this limitation. Second, the study is subject to selection bias due to the retrospective nature of the study and the subjectivity in categorizing the quality of frames. However, we reduced this by collecting data prospectively, curating test‐set II from consecutive cases and not restricting analysis to a single optimal image per polyp. Third, the CNN classification is limited to polyps and does not incorporate characterization of invasive malignancy, limiting its role to supporting ‘resect and discard’ and ‘diagnose and leave’ strategies. Fourth, evaluation in patient characteristics such as age and sex were not performed. Lastly, whilst the histopathology was assessed by expert gastrointestinal histopathologists, a consensus diagnosis would be more robust given the interobserver variability in differentiating SSL and hyperplastic polyps.

In conclusion, we demonstrated that a single CNN can differentiate adenoma polyps from nonadenomas in both NBI and NBI‐NF. A robust, publicly accessible NBI polyp video database was curated and benchmarked. Further evaluation in a clinical trial is warranted.

## CONFLICT OF INTEREST

Author L.B.L is the chair for the British Society of Gastroenterology Artifical Intelligence (AI) Task Force, a minor shareholder in Odin Vision, received research grants from Medtronic, Pentax Medical, and DynamX, and served on the Scientific Advisory Boards for Dynamx, Odin Vision, and Ninepoint Medical. R.K. is the secretary for the British Society of Gastroenterology AI Task Force. D.S. is a shareholder in Odin Vision and Digital Surgery Ltd. M.H. received speaker fees from Cook Medical. A.C.‐M., P.B., S.H., J.G.‐B.P., D.T., and P.M. are employees of Odin Vision Ltd. The other authors declare no conflict of interest for this article.

## FUNDING INFORMATION

This study was supported by the Wellcome/EPSRC Centre for Interventional and Surgical Sciences at UCL (203145Z/16/Z) and Innovate UK (26673). Author L.B.L. is supported by the National Institute for Health Research University College London Hospitals Biomedical Research Centre and the CRUK Experimental Cancer Medicine Centre at UCL.

## Supporting information


**Appendix S1.** Supplementary material.
