# Supplementary material for: Polyp characterization using deep learning and a publicly accessible polyp video database
Source: Dig Endosc. 2023 Jan 18;35(5):645–55. doi: 10.1111/den.14500 (PMC10570984; doi:10.1111/den.14500)
Supplement: Supplementary file 1 — Appendix S1. Supplementary material. [file DEN-35-645-s001.docx]

**SUPPLEMENTARY MATERIAL**

**Ethics**

The study was approved by the Cambridge central research medical ethics committee (REC Reference No. 18/EE/0148).

**Algorithm development**

**Data processing and annotation**

For each procedure, all video frames where a histologically confirmed polyp was visualised in NBI or NBI-NF was extracted and uploaded to a customised computer vision annotation tool (Figure S1) without alteration of the images. The videos generate 25 frames per second and inevitably capture frames with motion artifact due to the relative motion between the recording device and the position of the endoscope or when the endoscope is out of focus.^26^ A gastroenterology endoscopy fellow (R.K.) with expertise in optical diagnosis reviewed each individual frame to classify the image quality (Figure S2). If there was uncertainty then consensus was obtained by a further opinion from another endoscopist (L.B.L, R.V, E.S). High-quality frames were defined as images with a clear visualisation of the polyp. Moderate-quality frames were defined as the presence of artefact overlying the polyp (e.g. stool, blood, halation) or motion artefact that did not preclude visualisation of the polyp surface. Low-quality frames were defined as image blurriness, motion artifact, extremely dark images, or overlying polyp artefact that prevented visualisation of the polyp surface^15^. To avoid training the CNN with uninformative data (similar to previous protocols)^15^, low-quality frames were excluded. Moderate and high-quality frames were labelled with the imaging modality (NBI or NBI-NF), a bounding box around the polyps and the corresponding histology (Figure S2). These annotations were referenced as the ground truth.

**Data pre-processing and augmentation techniques**

Each input image is pre-processed before training or testing. The pre-process consists of a cropping based on the polyp box and a resizing of the resulting image to the model input size (224x224). During training, two types of augmentation techniques were performed: random affine transformations (translation, rotation, and scaling) and random colour transformations (brightness, contrast, and saturation). Moreover, the polyps were modified by randomly increasing their sizes adding between 0 and 200 pixels in each direction.

**Hyperparameters and training:**

The model was trained for 50 epochs with a batch size of 64. During each epoch 1024 frames equally balanced between adenomas and non-adenomas are sampled. The model weights were initialized with backbone weights from ImageNet. As an optimizer we have used AdaGrad with a learning rate of 0.0001. The final prediction score is obtained by applying a Softmax function to the last layer logits which in our case are two, one for each class (adenomas and non-adenomas).

**Model development:**

The model was trained with Pytorch on an NVIDIA GeForce RTX 2080 Ti Graphics Processing unit and followed a Resnet101 architecture (He et al., 2015) where the input is an image of size 224x224, and the format of the CNN output is a prediction score (0.0 – 1.0) with a threshold within this range differentiating adenomatous and non-adenomatous polyps. In Figure S3, a Resnet101 architecture is detailed. Each convolutional layer is composed of a convolutional layer of a kernel size nxn followed by a batch normalization layer and a RELU activation function. Each Residual block contain a skip connection, which means that the input of the block is added into the output of its last convolutional layer.

**Benchmarking of test-set II**

Three BCSP endoscopists with more than 10 years of colonoscopy experience (ADR >45%) and three JAG accredited independent endoscopists were enrolled in the web-based test. For the experiment, each polyp was assessed by the CNN, and by three expert (BCSP) and three non-expert (JAG accredited independent) endoscopists. For the endoscopists experiment, the same NBI video recording from test-set II was used, inclusive of all quality of frames, with the same image resolution (1920 x 1072). The web-based test was performed on a retina display computer via an online survey. The endoscopists evaluated each polyp video to determine whether the polyp in question was an adenoma or non-adenoma (hyperplastic/SSL) and recorded the confidence of their diagnosis (high or low confidence). Endoscopists were blinded to the polyp histology and permitted to watch each video sequence up to 3 times. The results are available in Table 4.

**Definition of performance metrices**

The following performance metrics were used for each frame:

True positive (TP) = CNN correctly diagnoses an adenomatous polyp as ‘adenoma’. The exception to this is the analysis of each polyp category (adenoma, hyperplastic, SSL) (Table 4) where TP refers to the histology of the polyp category evaluated.

False positive (FP) = CNN incorrectly diagnoses a hyperplastic or SSL polyp as ‘adenoma’.

True negative (TN) = CNN correctly diagnoses a hyperplastic or SSL polyp as ‘non-adenoma’

False negative (FN) = CNN incorrectly diagnoses an adenoma as ‘non-adenoma’. The exception to this is the analysis of each polyp category (adenoma, hyperplastic, SSL) (Table 4) where FN refers to the incorrect two polyp categories according to the ground truth histology.

Per frame sensitivity = TP / (TP + FN)

Per frame specificity = TN / (TN + FP)

Per frame accuracy = (TP+TN) / (TP + TN + FN + FP)

**Algorithm performance**

**Mean computation time:**

Mean computation time is strongly influenced by the performance of the GPU and CPU. In this study, the mean time for the analysis of each image was 0.014 seconds per image (+/- 0.004) for test set I, 0.015 seconds per image (+/- 0.008) for test set II and 0.014 (+/- 0.004) for the WEISS database (Table S1).

**CNN predictions of polyp frames on a per polyp basis**

For test-set I, 76% of polyps (n=119) were correctly characterised in 80% or more frames for each polyp, 15% of polyps (n=25) correct in 50 – 79% of frames, and 9% of polyps (n=14) correct in less than 50% of frames (Figure S4).

For test-set II, 78% of polyps (n=51) were correctly characterised in 80% or more frames for each polyp, 11% of polyps (n=7) correct in 50 – 79% of frames, and 11% of polyps (n=7) correct in less than 50% of frames.

**Median CNN prediction score:**

On a per-frame analysis, the median CNN prediction score for adenomas in test-set I and test-II were 0.995 (IQR 0.886 – 0.999) and 0.995 (IQR 0.914 – 0.999) respectively. For non-adenomas, these were 0.028 (IQR 0.001 – 0.163) and 0.010 (IQR 0.002 – 0.316).

On a per-polyp analysis, the median CNN prediction score for each polyp was 0.7 or above for 85% of adenomatous polyps in test set I and 88% in test set II. For non-adenomatous polyps, this was 0.49 or below for 87% of non-adenomatous polyps in test set I and 85% in test set II (Figure S5).

**Publicly accessible WEISS-database (test-set II)**

The polyp videos and associated meta-data, including polyp histology, location, size, and morphology, have been made publicly accessible to researchers. To obtain access to the database, called Wellcome/EPSRC Centre for Interventional and Surgical Sciences database (WEISS-database), researchers should email l.lovat@ucl.ac.uk. Upon agreeing with the terms of use, an electronic weblink will be sent by email to download the videos and meta-data.

**Figure S1:** Illustration of computer vision annotation tool





This customised tool was used to upload videos and annotate data. Annotations can be in the form of an image level label (e.g. imaging modality or quality of the image) or a bounding box around polyps.

**Figure S2:** NBI and NBI-NF imaging


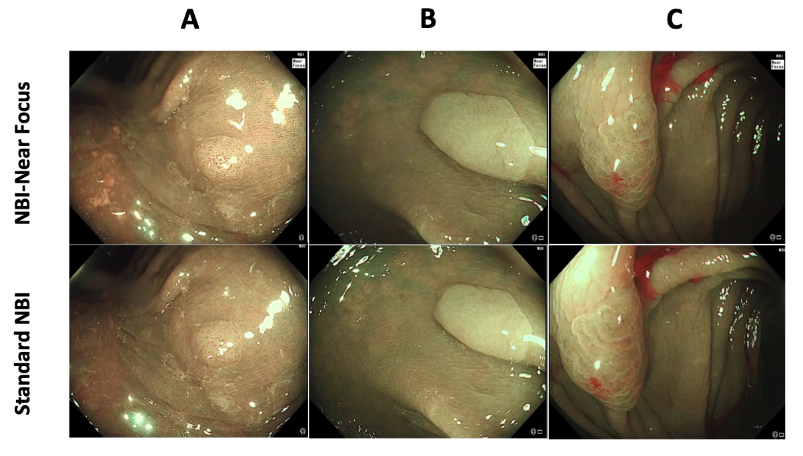


The same polyps visualised in both standard NBI and NBI-near focus (NBI-NF), demonstrating the closer observation of the polyp surface pattern with NBI-NF. (A) tubular adenoma (B) hyperplastic (C) sessile serrated lesion.

**Figure S3:** Resnet101 architecture


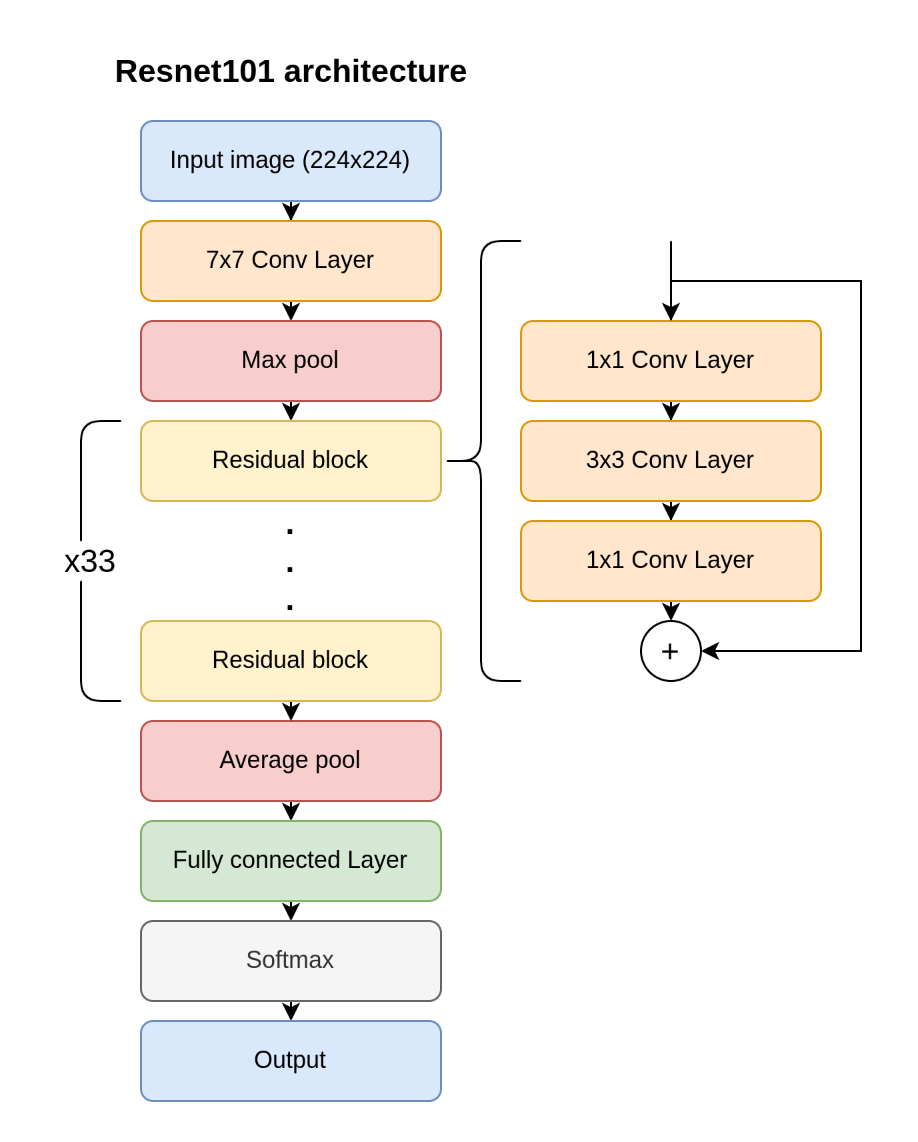


**Figure S4:** Proportion of frames correctly characterised for each polyp


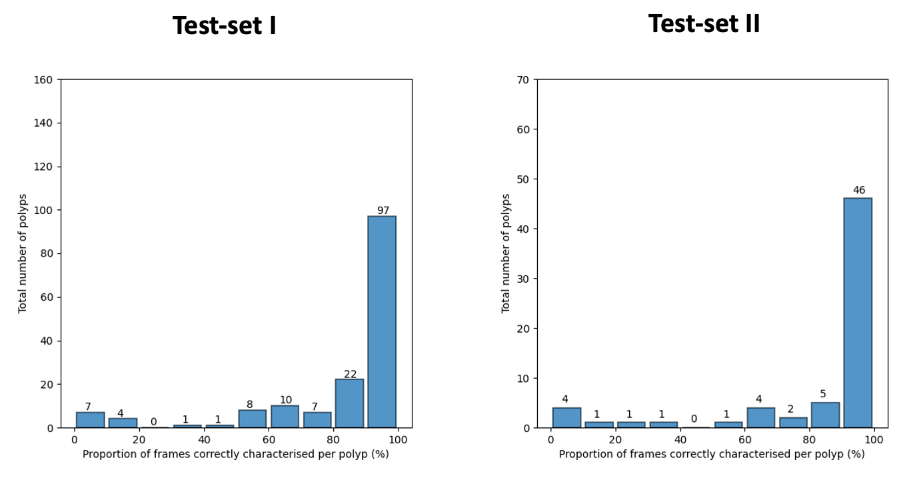


Results presented separately for test-set I (n=157) and test-set II (n=65).

**Figure S5:** Median CNN prediction score


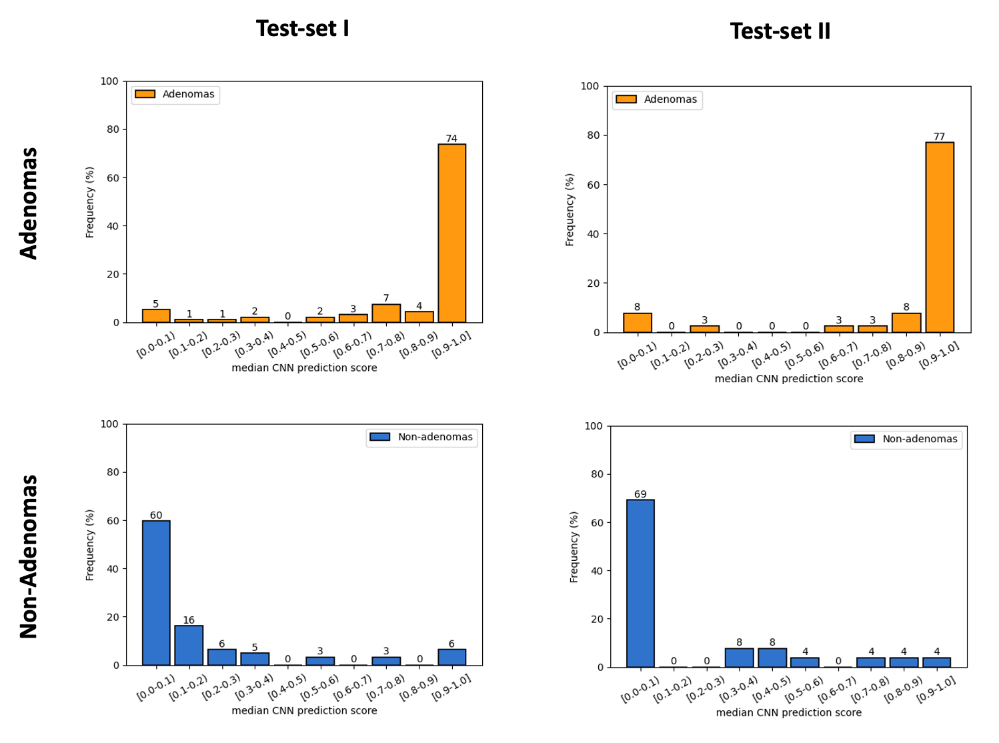


The distribution of the median CNN prediction score for each category of polyps (adenoma vs non-adenoma) in test set I and II.

**Table S1:** The mean CNN computation time to analyse video frames for test set I and test set II (+/- standard deviation).

| **Dataset** | **Mean computation time per image (seconds)** |
| --- | --- |
| **Test-set I** | 0.014181 ± 0.003671 |
| **Test-set II** | 0.015197 ± 0.007928 |

The mean computation time for the CN to analyse video frames for test set I and test set II (+/- standard deviation). CNN = convolutional neural network
